# The Spatial Overlap of Police Calls Reporting Street-Level and Behind-Closed-Doors Crime: A Bayesian Modeling Approach

**DOI:** 10.3390/ijerph18105426

**Published:** 2021-05-19

**Authors:** Miriam Marco, Enrique Gracia, Antonio López-Quílez, Marisol Lila

**Affiliations:** 1 Department of Social Psychology, University of Valencia, 46010 Valencia, Spain; enrique.gracia@uv.es (E.G.); marisol.lila@uv.es (M.L.); 2 Department of Statistics and Operational Research, University of Valencia, Burjassot, 46100 Valencia, Spain; antonio.lopez@uv.es

**Keywords:** joint modeling, Bayesian spatial analysis, intimate-partner violence, street-level crime, violence behind closed doors, neighborhoods

## Abstract

Traditionally, intimate-partner violence has been considered a special type of crime that occurs *behind closed doors*, with different characteristics from street-level crime. The aim of this study is to analyze the spatial overlap of police calls reporting street-level and behind-closed-doors crime. We analyzed geocoded police calls in the 552 census-block groups of the city of Valencia, Spain, related to street-level crime (*N* = 26,624) and to intimate-partner violence against women (*N* = 11,673). A Bayesian joint model was run to analyze the spatial overlap. In addition, two Bayesian hierarchical models controlled for different neighborhood characteristics to analyze the relative risks. Results showed that 66.5% of the total between-area variation in risk of reporting street-level crime was captured by a shared spatial component, while for reporting IPVAW the shared component was 91.1%. The log relative risks showed a correlation of 0.53, with 73.6% of the census-block groups having either low or high values in both outcomes, and 26.4% of the areas with mismatched risks. Maps of the shared component and the relative risks are shown to detect spatial differences. These results suggest that although there are some spatial differences between police calls reporting street-level and behind-closed-doors crime, there is also a shared distribution that should be considered to inform better-targeted police interventions.

## 1. Introduction

Spatial modeling is an advanced methodological approach that is increasingly being used in the study of crime. Previous research has spatially analyzed different types of crime, focusing on the prediction of crime, detection of spatial clusters and hotspots, and the neighborhood-level characteristics related to crime risk [1,2,3]. These studies found that crime is not distributed randomly in cities but shows spatial patterns, which in turn are related to the characteristics of the neighborhoods where it occurs [4,5,6,7,8].

One of the main methodological advances in recent years in the spatial analysis of crime is the application of Bayesian spatial modeling [9]. These models improve on other models by allowing researchers to analyze spatial structures and to assess and map specific area risks [9]. They can also be used to address autocorrelation and heterogeneity issues [5,10], and are particularly suitable for small-area spatial analysis [5,11].

Particularly relevant is the study of street-level crime using Bayesian spatial modeling. Street-level crime refers to any criminal offense occurring in a public place. Following this approach, a large number of studies have explored the spatial distribution of criminal activities such as violent crime and homicides [8,12,13,14], drug-related outcomes [15,16], burglary [17,18,19,20], and juvenile delinquency [21]. Previous findings in the literature showing that community-level characteristics are involved in explaining crime beyond the individual level [22,23] were also supported by these Bayesian spatial studies, advancing the knowledge base in this research field.

Few studies, on the other hand, have focused on the spatial patterns of violence behind closed doors, such as intimate-partner violence [24,25,26,27], and child abuse and neglect [28,29,30,31,32,33]. Traditionally, intimate-partner violence (IPV) has been considered a special type of crime that occurs behind closed doors, with different characteristics from street-level crime. Unlike street-level crime, some scholars have considered IPV relatively impervious to contextual factors because of the private environment in which it generally occurs [34]. In addition, the role of social processes in explaining the link between communities and street-level crime, such as informal social control or collective efficacy [23,35], has been debated in the case of IPV [36,37]. If the incidents take place within the privacy of the home, away from the scrutiny of neighbors and community systems, individual and relational factors will probably have more weight in explaining IPV, thus limiting the effect of contextual factors [34].

To examine the link between community factors and IPV with appropriate methodologies, a limited number of studies have assessed the spatial distribution of IPV and its contextual determinants using Bayesian spatial modeling [24,25,26,38,39]. This body of research supports the view that place matters, and that neighborhood characteristics are relevant to explain IPV risk variations. Among these community characteristics, neighborhood disadvantage––measured by means of poverty, unemployment, education, or neighborhood disorder––emerges as a particularly relevant predictor of spatial inequality in IPV risk [24,25,26,38].

This body of research shows that violence and crime, whether occurring behind closed doors or in the streets, are not randomly distributed in cities, and highlights the need for advanced spatial methodologies to better understand their links. In this regard, new methodological approaches have emerged in the past years. For example, some studies have explored the common spatial patterns of different outcomes related to crime using multivariate spatial techniques [40,41,42,43]. These models aim to understand separately the potentially common spatial component and the two specific spatial patterns [43]; thus, they allow joint assessment of the spatial distribution of two or more types of crime [40,44,45]. In the context of violence behind closed doors, some studies have analyzed the common spatial distribution of child maltreatment and intimate-partner violence [44], as well as the spatial overlap of substantiated and unsubstantiated child-maltreatment referrals [45]. However, to the best of our knowledge, no previous studies have assessed the joint spatial distribution of street-level crime and one type of behind-closed-doors crime, namely intimate-partner violence against women (IPVAW). This approach could provide more information on the spatial relationship of the two types of crime. To this end, in the present study we will analyze the spatial overlap of police calls reporting street-level crime and IPVAW.

## 2. Materials and Methods

### 2.1. Data and Study Area

The study area was the city of Valencia, Spain. Valencia is one of the largest cities in Spain, with a population of 800,215 inhabitants (2020 data) and a population density of 5850 inhabitants per square kilometer. The city’s 552 census-block groups were used as the area unit for the study. This is the smallest unit with available administrative data, so it was considered as a proxy for neighborhoods.

The outcomes (street-level and violence-behind-closed-doors crime) were measured as the police calls reporting these types of crimes received by the Valencia Police Department. The Valencia Police Department provided data of all police calls for service made from 2010 to 2016 in the city of Valencia typified as *citizen security, offense against the person*. The two different types of police calls analyzed were: (1) street-level crime: fights (28%) and assaults (72%), with a total of *N* = 26,624, and (2) behind-closed-doors crime: police calls requiring service to help a woman who is a victim of a current or former male partner, typified as IPVAW (*N* = 11,673). Other types of intimate-partner violence, such as violence perpetrated by a woman to her male partner, or violence between same-sex couples were excluded for two main reasons: first, the proportion of these cases is very small compared to IPVAW cases, and second, Spanish legislation classifies intimate-partner violence into two different typologies, IPVAW (which has a special police and judicial protection), and domestic violence, which includes any other type of violence behind closed doors, such as violence between any other two partnered people, but also child maltreatment, child-parent violence, or violence between other family members. Police-call classification is based on this typology, and thus, incorporating domestic violence would add biases due to the heterogeneity of this category.

Data incorporated the coordinates where the incidents took place. The calls were geolocated and counted on the 552 census-block groups. In addition, census-block group level variables set by the City Hall were used as control variables (see Table 1).

*Mean income*: The average mean income per person (in euros).

*Education level*: The average education level was measured on a scale from 0 to 4, where 1 = less than primary education, 2 = primary education, 3 = secondary education, 4 = college education.

*Physical vulnerability*: Index calculated using various indicators of the difficulty in accessing public resources, including hospitals and healthcare centers, transport, schools, social services, sports centers, police stations, libraries, and parks. The index ranged from 1 (low vulnerability) to 5 (high vulnerability).

*Physical disorder and decay*: Trained raters assessed the level of disorder using a systematic social observation scale [46]. Two different indicators were used: physical disorder, which included eight items such as cigarettes and trash, or empty bottles in the street, graffiti or abandoned cars; and physical decay, composed of four items, namely vacant houses; abandoned, vandalized and rundown buildings; deteriorated residential units, and deteriorated recreation places. Each item was rated from 0 to 4, where 0 indicates no presence and 4 indicates high presence.

*Vacant lots:* Percentage of vacant lots in the total area.

*Immigration*: Percentage of immigrant population.

*Residential instability*: The proportion of the population who had moved into or out of each census-block group during the previous year per 1000 inhabitants.

*Alcohol outlet density*: Three categories of alcohol outlets were used [47], off-premise outlets (retail sale of food, wines and beverages), restaurants/cafés (services in restaurants and coffee shops), and bars. The density was calculated as the number of establishments per square kilometer.

### 2.2. Data Analysis

#### 2.2.1. Joint Spatial Modeling

First, a Bayesian joint model was run to analyze the spatial overlap of police calls reporting street-level crime and IPVAW. We assumed that both outcomes followed a conditional independent Poisson distribution: they are assessed as counts of police calls in each area, and these counts are assumed to be independent of one another. The joint model proposed by Knorr-Held and Best [48] was conducted. This model follows an epidemiological approach and divides the spatial distribution into a shared component as well as two specific components for each outcome. Equation (1) shows the structure of the model:

(1) $$Y_{ik}\sim Po\left(\mu_{ik}\right)\;\log\mu_{i1}=\log E_{i1}+\alpha_{1}+\varphi_{i}*\delta +\psi_{i1}\;\log\mu_{i2}=\log E_{i2}+\alpha_{2}+\varphi_{i}/\delta +\psi_{i2}$$

where $Y_{ik}$ represents the observed counts for outcome *k* (1 for police calls reporting street-level crime and 2 for reporting IPVAW) in census block group *i*; $\mu_{ik}$ is the unknown mean; $E_{ik}$ is the expected counts for each outcome k in census block group i; $\alpha_{¡i}$ is the intercept; δ is the scaling factor that accounts for the gradient in which the shared component (represented by φ) is different for each outcome, i.e., as the magnitude of the area-specific relative risks may differ, δ controls and rescales the relative risks to avoid this possible difference; and $\psi_{ik}$ accounts for the two specific components. Finally, φ and ψ are composed of structured and unstructured spatial parameters, i.e., the spatial autocorrelation and the spatial heterogeneity, respectively, following a conditionally autoregressive (CAR) model [49]. The percentage of shared variance was calculated for each outcome ($\eta_{k}$).

#### 2.2.2. Spatial Regression Modeling

Once the spatial overlap was assessed using the joint modeling, two Poisson Bayesian hierarchical models were conducted for each outcome [49]. This modeling allows incorporation of neighborhood-level covariates as fixed effects, but generally it also includes two spatial random effects: a spatial term that controls for the spatial autocorrelation, and a spatial heterogeneity to avoid biases related to data overdispersion. These two models are useful to understand the relationship between the outcomes and the contextual characteristics, but they were especially used to measure the relative risks of each outcome and compare the areas of high risk to assess and map the spatial similarity between reporting street-level crime and reporting IPVAW. The models were defined as follows in Equation (2):

(2) $$\log\mu_{ik}=\log E_{ik}+\alpha_{k}+X_{i}\beta_{k}+\phi_{i}+\theta_{i}$$

This model incorporates the spatial autocorrelation and the spatial heterogeneity (ϕ and θ, respectively), as well as different covariates represented by β as the regression coefficients vector and X as the matrix of covariates. The covariates were introduced to control for neighborhood characteristics (mean income, education level, physical vulnerability, physical disorder and decay, vacant lots, immigration, residential instability, and alcohol outlet density). The relative risks were analyzed as in Equation (3):

(3) $$\;\eta_{ik}=\exp\left(\alpha_{k}+X_{i}\beta_{k}+\phi_{i}+\theta_{i}\right)$$

In addition, the Pearson correlation coefficient was calculated and represented in a scatter plot to assess the association between them. Finally, the map of coincident relative risks showed the areas with coincident above-average and below-average levels of relative risk for both outcomes.

#### 2.2.3. Bayesian Inference

Following a Bayesian approach, the parameters were considered random variables, and we assigned prior distributions for each parameter. Specifically, we modeled α using an improper uniform distribution (which can take any real value), and the fixed effects β were modeled as vague Gaussian distributions (i.e., normal distributions with a mean of 0 and variance of 100.000). These distributions are uninformative and allow results to be mainly based on the data.

The spatial parameter θ (identifying the unstructured spatial term) was modeled as Gaussian random variables $N\left(0,\sigma_{\theta }^{2}\right)$, and ϕ (the structured spatial effect) was modeled as a conditional spatial autoregressive (CAR) model [49], following Equation (4):

(4) $$\left.\phi_{i}\right|\phi_{-i}\sim N(\frac{1}{n_{i}}\underset{j\sim i}{\sum }\phi_{j},\frac{\sigma_{\phi }^{2}}{n_{i}})$$

where $n_{i}$ is the number of neighboring areas of census block group i; $\phi_{-i}$ indicates the values of the ϕ vector except the i-th component; the expression j~i denotes all units j that are neighbors of census block group i; and $\sigma_{\phi }$ is the standard deviation parameter. The CAR model is the most common distribution used for modeling spatial dependence when analyzing spatial areas such as census-block groups.

In addition, we assigned a uniform distribution $U\left(0,2\right)$ as hyperpriors for the hyperparameters $\sigma_{\phi }$ and $\sigma_{\theta }$. In a sensitivity analysis varying the priors of the model, similar results were found.

All Bayesian models were fitted using the Markov Chain Monte Carlo (MCMC) simulation approach [50]. In each model, 100,000 iterations were generated, with a burn-in period of 10,000 iterations. The convergence diagnosis $\hat{R}$ was near 1.0 for all parameters, indicating a good convergence. The analyses were conducted using the statistical software R, and the Bayesian simulations were performed using WinBUGS software (the BUGS Project, London, UK).

## 3. Results

### 3.1. Spatial Joint Modeling

Table 2 shows the results of the posterior distribution of the parameters of spatial joint modeling. Results showed that 66.5% of the total between-area variation in risk of reporting street-level crime was captured by a shared spatial component, while for reporting IPVAW the shared component was 91.1%. This result indicates that the main common spatial pattern is provided by reporting IPVAW, while reporting street-level crime has a large part of its variability (33.5%) explained by its specific component. The scaling factor δ shows a value close to 1, indicating that the magnitude of the relative risks for both outcomes are the same.

**Figure 1:**
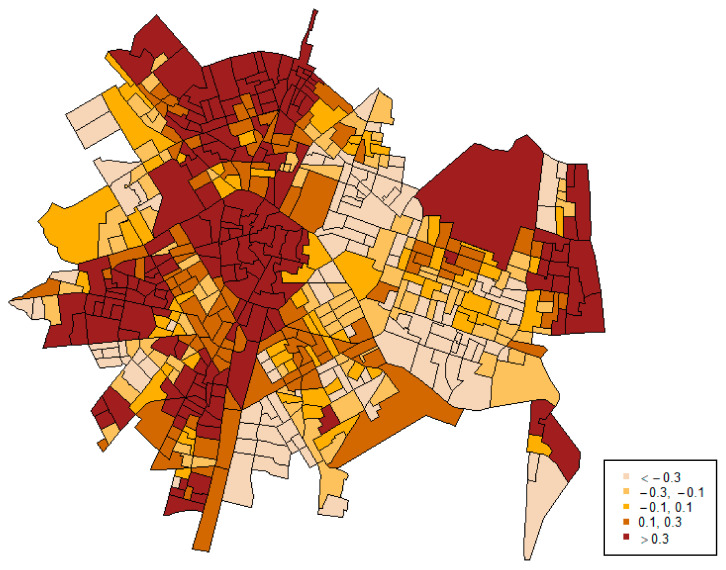
Shared spatial component.

Figure 1 represents the map of the shared component. It is based on the logarithm of the relative risk, so values can range from negative to positive numbers. Its interpretation is not directly associated with the scale and the specific values, but it is comparative. Areas with higher positive values of the shared component (the darker areas) would show a higher relative risk (in this case, a higher joint prevalence of both outcomes), while areas with lower and negative values (the lighter areas) would indicate a lower level of relative risk (a lower joint prevalence of them).

This map shows a spatial pattern according to which the central areas of the city, as well as some peripherally concentrated areas in the north, the west and the east (on the coast) show higher prevalence of both types of police calls.

### 3.2. Spatial Regression Modeling

As the spatial joint modeling showed a common spatial pattern between police calls reporting street-level crime and IPVAW, it was considered appropriate to conduct two different Bayesian Poisson spatial regression models and analyze the correlation between their relative risks. Table 3 shows the results of the two models, including spatial parameters and control covariates.

Several associations were found between the outcomes and the control covariates, represented by $\beta_{k}$ (the regression coefficients) in Equation (2). Specifically, and following the criterion of an above 80% probability of being positive or negative [26] (i.e., considering as relevant those covariates with a posterior probability of being positive or negative higher than 80%), reporting street-level crime was related to areas with higher mean income, higher immigration, higher physical disorder and vacant lots, higher residential instability, and higher bar density. Reporting IPVAW, however, was greater in areas with low levels of education, higher physical vulnerability, higher percentage of immigration, higher physical disorder, and higher bar density.

Regarding the spatial effects, the mean value for the standard deviation of the spatial structured term $\sigma_{\phi }$ (the spatial autocorrelation) is higher than the standard deviation of the spatial unstructured term $\sigma_{\theta }$ (the spatial heterogeneity), which means that the first is contributing to a greater extent than the second, but both have a strong weight in the model.

**Figure 2:**
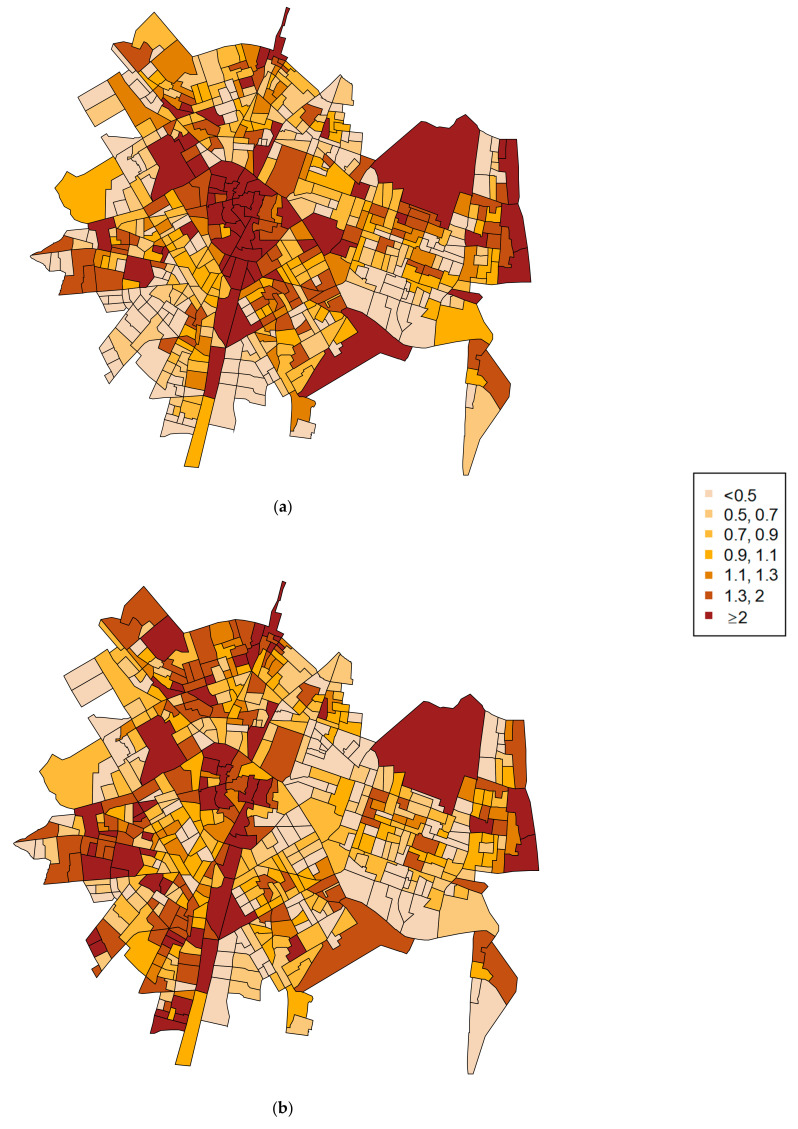
Relative risk for (**a**) police calls reporting street-level crime and (**b**) police calls reporting violence behind closed doors (IPVAW).

The maps of relative risk are presented in Figure 2. The relative risk values were calculated as $\eta_{ik}$ as shown in Equation (3). With 1 being the average risk, areas with values lower than 1 represent a below-average relative risk (the lighter areas in the map), while areas with values indicate an above-average relative risk (the darker areas). Specifically, areas with values over 2 would mean a relative risk twice the average.

We found areas with similar patterns for both outcomes, as highlighted by the shared spatial component. However, we also detected specific local areas with notable differences in the relative risk. For example, the census-block groups located in the south of the city present high levels of IPVAW calls but low levels of calls reporting street-level crime.

**Figure 3:**
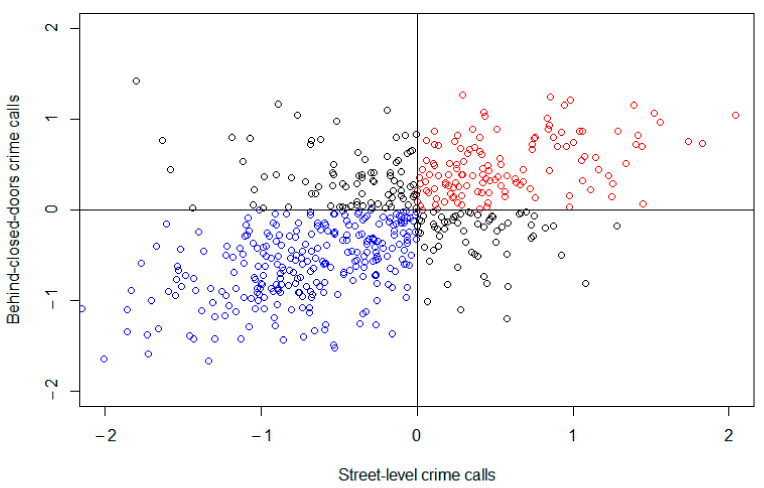
Scatter plot of the correlation between log relative risk for street-level and behind-closed-doors crime calls.

We calculated the Pearson correlation coefficient to obtain a better measure of coincidence between risks; Figure 3 shows the scatter plot for this relationship. The results showed a correlation of 0.53, with many census-block groups matching in low-risk area (blue) and high-risk area (red), but there was also a considerable number of census-block groups with mismatching risks (black).

**Figure 4:**
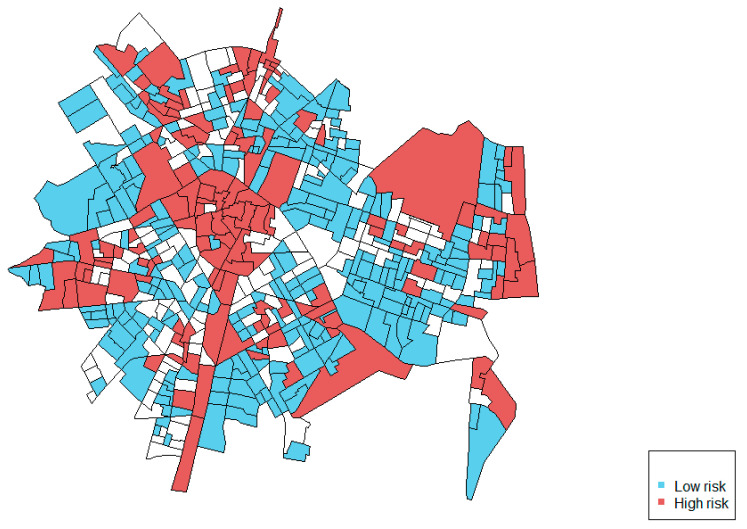
Map of coincident risk areas.

Figure 4 shows the map of the coincident areas, showing the areas with higher than average (red) or lower than average (blue) relative risks for police calls reporting both street-level crime and IPVAW. Results show 73.6% of the census-block groups either with low or high values in both outcomes, and 26.4% of the areas with mismatched risks.

## 4. Discussion

This study aimed to analyze whether there was a shared spatial distribution of police calls reporting street-level crime and IPVAW. A Bayesian joint model was performed in line with previous research that incorporates a multivariate spatial analysis to study crime outcomes [40,41,42,43,51,52]. In addition, two different Poisson regression models were conducted to assess the spatial similarity of relative risks.

Two key findings emerged from our study: first, there was a shared distribution between the two types of crime outcomes. Specifically, results showed that 66.5% of the variation in risk of police calls reporting street-level crime was captured by a shared spatial component, while for reporting IPVAW, 91.1% of the variation across city areas was explained by this shared component. This finding indicates that the shared spatial component captured most of the spatial variability of police calls reporting IPVAW; that is, most of the spatial pattern of reporting IPVAW is shared with the spatial pattern of reporting street-level crime. The map of the shared spatial term suggests that the spatial pattern is especially intense in the city center. In addition, we detected a common pattern that separates the western (with higher risks for both outcomes) and the eastern parts of the city (with lower risks), added to another above-risk area along the coast in the east.

The second finding shows that despite sharing these common spatial patterns, there were also some spatial differences between police calls reporting street-level crime and IPVAW. On the one hand, the joint modeling shows a relevant percentage of area variation for reporting street-level crime that is not explained by the shared component (33.5%). This suggests that there is also a crime-specific spatial pattern related to reporting street-level crime that differs from that for police calls reporting IPVAW. The maps of the relative risks show this difference and identify specific areas where the relative risk was higher for street-level crime. The correlation of the relative risks (0.53), in addition, shows that 73.6% of the areas had coincident low or high risk in both types of crime (street-level and behind-closed-doors), but the other 26.4% of the census-block groups had mismatched risks (with a low risk for one outcome and high risk for the other).

These results suggest that some areas of the city present higher levels of police calls regardless of the type of crime (street-level or behind-closed-doors). These areas would require more police resources to attend to the needs of citizens. Other areas, nevertheless, differ in both types of calls, especially those areas with a higher prevalence of police calls for street-level crime only. The prevention and intervention strategies should be sensitive to these localized differences, in order to design better-targeted police interventions.

There is a large tradition in the study of the spatial structures of crime using modeling approaches from a non-Bayesian perspective. This research has explored the spatial distribution of crime following different criminological theories such as crime pattern theory [53,54], social disorganization theory [55,56], near-repeat victimization patterns modeling [57,58,59], and routine activities theory [60,61]. In addition, other methods, especially from geostatistics, such as Kernel Density Smoothing (KDS) [62]; or Risk Terrain Modeling [63,64,65] have been used to analyze, visualize, and predict crime data. These methods have contributed to demonstrating that crime is not randomly distributed in the cities; there are notable patterns that should be analyzed to acquire a greater comprehension of crime processes and prediction.

However, Bayesian spatial modeling has proven some advantages, especially related to a better control of biases such as autocorrelation, ecological fallacy, and heterogeneity issues [5,10], which is translating to an increasing use of this methodology for the analysis of crime [8,14,25,26]. This study provides more insights about the spatial distribution of reported street-level and behind-closed-doors crime and its spatial similarity from a Bayesian perspective, a topic where there is less research.

Our study has some limitations that should be kept in mind. First, crime was measured using police calls. Although reporting crime and police calls are commonly used, especially for street-level crime [18,42], this measure may have intrinsic biases due to the possible falsehood of the crime event or the misinterpretation of the crime situation when a witness informs the police. In addition, police calls do not distinguish between single and multiple calls referring to the same incident and/or the same actors, which leads to a possible data dependency bias [24]. Other useful measures such as police-report records of criminal offenses [21,24,41,66], assaults resulting in hospitalizations [12], IPV-related medical records and hospitalizations [38], and self-reported IPV were not available. In addition, violence behind closed doors was measured using IPVAW; however, other IPV types, such as violence perpetrated by female partners or same-sex couples were not incorporated in this analysis, and thus the results are not generalizable to those types of IPV.

Second, as this study only assessed the shared spatial patterns, future studies would benefit from incorporating a spatio–temporal approach to avoid possible aggregation bias. Moreover, this study used census-block groups, which was the smallest spatial unit available, and it was considered that they better represent neighborhoods compared to larger units such as census tracts or zip codes. Notwithstanding, this aggregation could mask associations between the outcomes and the covariables due to the heterogeneity of areas. This study could be complemented using, when they are available, alternative and smaller units such as streets or street segments [67,68]. Third, this study was conducted in one Spanish city, but results may differ with samples from other countries and cultural contexts. Cross-cultural studies are needed to compare the findings and establish more robust conclusions.

## 5. Conclusions

This study addresses a shortcoming in the literature, as it analyzes the spatial overlap of police calls reporting street-level crime and a type of behind-closed-doors crime, IPVAW, using an advanced methodological approach––Bayesian spatial modeling. The findings suggest that police calls reporting street-level crime and IPVAW share some spatial structures, although this is a moderate relationship. Using Bayesian spatial models can help to better understand the spatial structures of both street-level crime and IPVAW, and can be a useful tool for crime monitoring and prevention.

## Figures and Tables

**Table 1:** Descriptive statistics for the outcomes and the control variables.

Variable	Mean	SD	(Min, Max)
Mean income (€)	12,285	4031.33	(5170, 29,364)
Education (0–4)	3.15	0.33	(2.39, 3.86)
Vulnerability (1–5)	3.07	0.34	(1.77, 3.95)
Physical disorder (0–32)	8.88	5.13	(0, 26)
Physical decay (0–16)	3.04	2.79	(0, 14)
Vacant lots (%)	1.03	3.11	(0, 63.71)
Immigration (%)	15.16	7.31	(3.13, 49.05)
Residential instability (per 1000 inhabitants)	229.70	91.18	(62.00, 606.00)
Off-premise density (per km^2^)	55.13	66.8	(0, 744.04)
Restaurant/café density (per km^2^)	48.83	72.2	(0, 581.66)
Bar density (per km^2^)	154.06	127.3	(0, 940.88)
Street-level crime calls (per 1000 inhabitants)	402.97	43.00	(2.23, 402.97)
IPVAW calls (per 1000 inhabitants)	16.19	11.40	(0.00, 67.46)

**Table 2:** Posterior distribution of the parameters of spatial joint modeling.

	Mean	SD	CrI 95%
$\alpha_{1}$	0.222	0.781	−0.46, 2.476
$\alpha_{2}$	0.27	0.749	−0.373, 2.42
δ	1.004	0.068	0.911, 1.192
$\eta_{1}$	0.665	0.124	0.542, 0.994
$\eta_{2}$	0.911	0.131	0.59, 0.999

CrI, Credible Interval; α, intercept; δ, scaling factor; η, shared variance; _1_ Police calls reporting street level crime; _2_ Police calls reporting IPVAW.

**Table 3:** Posterior distribution of the parameters of the spatial regression models.

	Street Level Crime		IPV Calls	
Variable	Mean (SD)	95% CrI	Mean (SD)	95% CrI
α	−0.977 (0.683)	−2.363, 0.369	−0.228 (0.585)	−1.311, 0.948
Mean income	0.449 (0.178)	0.108, 0.813	0.086 (0.191)	−0.287, 0.444
Education	−0.077 (0.206)	−0.498, 0.336	−0.256 (0.192)	−0.615, 0.146
Vulnerability	−0.005 (0.097)	−0.187, 0.188	0.078 (0.091)	−0.111, 0.253
Immigration	0.008 (0.009)	−0.010, 0.025	0.013 (0.009)	−0.003, 0.030
Physical disorder	0.011 (0.010)	−0.009, 0.031	0.010 (0.009)	−0.009, 0.027
Physical decay	0.009 (0.018)	−0.027, 0.044	0.009 (0.017)	−0.025, 0.043
Vacant lots	0.009 (0.009)	−0.009, 0.026	0.002 (0.009)	−0.016, 0.018
Immigration	0.008 (0.009)	−0.010, 0.025	0.013 (0.009)	−0.003, 0.030
Residential instability	0.001 (0.001)	0.000, 0.002	0.001 (0.001)	0.000, 0.002
Off-premise density	0.000 (0.000)	−0.001, 0.001	0.000 (0.000)	−0.001, 0.001
Bar density	−0.001 (0.000)	−0.001, 0.000	−0.001 (0.000)	−0.001, 0.000
Restaurants-cafés density	0.000 (0.000)	−0.001, 0.001	0.000 (0.000)	−0.001, 0.001
$\sigma_{\phi }$	0.889 (0.189)	0.567, 1.245	0.683 (0.118)	0.470, 0.941
$\sigma_{\theta }$	0.441 (0.074)	0.297, 0.569	0.460 (0.045)	0.365, 0.541

CrI, Credible Interval; SD, standard deviation; α, intercept; $\sigma_{\phi }$, standard deviation spatially structured term; $\sigma_{\theta }$, standard deviation spatially unstructured term.

## Data Availability

Restrictions apply to the availability of the data. Data were obtained from Valencia Police Department and are not publicly available due to confidentiality issues.
